# Medicine’s beauty lies in lifelong learning, reflection and research

**DOI:** 10.1016/j.clinme.2025.100516

**Published:** 2025-09-24

**Authors:** Ilfita Sahbudin, Ponnusamy Saravanan

**Affiliations:** a Rheumatology Research Group, Department of Inflammation and Ageing, College of Medicine and Health, University of Birmingham, Birmingham, UK; b National Institute for Health and Care Research (NIHR) Birmingham Biomedical Research Centre, University Hospitals Birmingham NHS Foundation Trust, Birmingham, UK; c Warwick Medical School, Warwick Applied Health, University of Warwick, Coventry, UK; d Centre for Global Health, University of Warwick, Coventry, UK; e Diabetes, Endocrinology and Metabolism, George Eliot Hospital NHS Trust, Nuneaton, UK

I always look forward to the time of introducing the latest issue of Clinical Medicine. This one contains several exciting CME topics on rheumatology. It’s a pleasure and a privilege to write a joint editorial, this time with Dr Ilfita Sahbudin to highlight the importance of specialty medicine for the general medical audience. I am grateful to Dr Sahbudin for her careful selection of rheumatology topics, which are highly relevant to generalists. Rheumatology holds a special place in my heart; in my view, it was one of the first few specialties to translate exciting new advances in discovery science into clinical practice, such as the initial use of monoclonal antibodies. This issue offers valuable learning insights for clinicians and academics across all fields of medicine. Summaries of these articles are included below, along with highlights of two original research papers and an article that summarises two cases on POEMS syndrome – my picks for this issue.

Gout is the oldest rheumatic disease. ‘Podagra’, acute gout of the big toe, was first reported by the Egyptians around 2640 BCE. Hippocrates subsequently described this malady as the ‘unwalkable disease’ in the 5th century BCE.^1^ Fast forward to 2025, Abhishek *et al* provide modern know-how for general physicians on managing this disease on the medical wards.^2^ This article also highlights the choice of anti-inflammatory medications to treat gout flare with special consideration for specific patient populations, such as patients with chronic kidney disease and heart failure.

Immune checkpoint inhibitor-induced inflammatory arthritis is the latest clinical entity in rheumatology. Immune checkpoint inhibitors (ICI) are a type of immunotherapy developed for cancer treatment, with the first agent (ipilimumab) approved in 2011.^3^ As ICI use became more common in oncology, rheumatologists began reporting associated inflammatory arthritis in patients undergoing ICI therapy for cancer treatment.^4^ This phenomenon highlights how therapeutic advancement in one medical specialty led to a new evolving disease that requires management by another medical specialty. Fisher *et al* provide an overview of the clinical characteristics, investigations and management of ICI-induced inflammatory arthritis.^5^

Systemic lupus erythematosus (SLE) diagnosis can be a challenge. The natural history of SLE with non-specific signs and symptoms during early disease, coupled with the heterogeneity of SLE clinical features and a low-specificity diagnostic test, often creates a diagnostic conundrum.^6^^,^^7^ Yusof *et al* provide some pragmatic guidance to general physicians when approaching a patient with suspected SLE.^8^

The treatment landscape in rheumatology has evolved rapidly since the first biologic for rheumatoid arthritis, infliximab, was approved in 2000. More recently, a new class of medication, Janus kinase (JAK) inhibitors, is increasingly prescribed in rheumatology. Filer *et al* provide a concise therapeutic update in three rheumatic diseases: rheumatoid arthritis, inflammatory idiopathic myositis and ANCA-associated vasculitis.^9^

New medication leads to new medication safety updates; JAK inhibitors confer additional risks of cardiovascular events and venous thromboembolism.^10^ In addition, broader differentials should be considered for patients on disease-modifying anti-rheumatic drugs (DMARDs) when they present with common symptoms. Croft and Singh have designed educational cases to highlight pertinent learning points when approaching rheumatology patients treated with DMARDs presenting in acute care.^11^

Alongside therapeutic advancements, there have also been important diagnostic developments in rheumatology. The availability of high-resolution ultrasound imaging tools enables rheumatologists to capitalise on this technology to improve clinical care. Sahbudin *et al* summarise how ultrasound imaging drives diagnostic and therapeutic decisions in rheumatology. This article also highlights the latest update in artificial intelligence (AI) in rheumatology ultrasound, and the current state of rheumatology ultrasound training in the UK.^12^

Finally, my selections for this issue. Manga *et al’*s work from South Africa highlights the rising burden of non-communicable disease in pregnancy. They present contemporary data on diabetes in pregnancy, including gestational diabetes and pregnancy outcomes. This underscores the value of prediction and risk stratification across different populations. Their findings highlight the need for population-specific approaches, though further evidence is required as this study is based on single-centre, hospital data.^13^ Palin *et al* provide a useful summary reminding us that infection remains a major cause of morbidity and mortality in heart failure. They explain why patients with heart failure are particularly susceptible to infection and stress the importance of early recognition and treatment.^14^ Lastly, Maikovsky and Williams describe two cases of POEMS (polyneuropathy, organomegaly, endocrinopathy, monoclonal gammopathy and skin abnormalities) syndrome, illustrating the poor outcomes that arise when rare but treatable multisystem conditions go unrecognised.^15^ The true beauty of medicine lies in lifelong learning and reflection.

## Declaration of competing interest

Dr Sahbudin is a member of the editorial board of *Clinical Medicine*. Prof Saravanan is the editor-in-chief of *Clinical Medicine*.
